# Exploring the relationship between condition severity and health-related quality of life in people with haemophilia A across Europe: a multivariable analysis of data from the CHESS II study

**DOI:** 10.1186/s12955-024-02267-6

**Published:** 2024-07-29

**Authors:** Enrico Ferri Grazzi, Charles Hawes, Charlotte Camp, David Hinds, Jamie O’Hara, Tom Burke

**Affiliations:** 1 HCD Economics, Keckwick Lane, Daresbury, Cheshire WA4 4FS UK; 2 BioMarin Europe, London, UK; 3 BioMarin Pharmaceutical, San Rafael, CA USA; 4 University of Chester, Chester, UK

**Keywords:** Haemophilia A, Patient-reported outcomes, Health-related quality of life, Pain, Annual bleeding rate, Problem joints, Severe haemophilia, Clotting factor VIII

## Abstract

**Background:**

Haemophilia A (HA; Factor VIII deficiency) is a congenital X-linked bleeding disorder characterized by trauma-related or spontaneous bleeding events, most notably arising within the intraarticular space and resulting in chronic inflammation and degeneration of affected joints. Endogenous clotting factor activity relative to normal levels determines the severity of HA symptoms, as mild (> 5–40%), moderate (1–5%), or severe (< 1%). Within the current environment of rapid evolution in HA management, we seek to understand the interplay of condition severity and health-related quality of life (HRQoL) to characterise and differentiate unmet needs among people with HA (PwHA).

**Methods:**

A generalised linear regression model (GLM) was developed to explore the relationship between HA severity and EQ-5D-5 L index score from adult HA patients sampled in the “Cost of Haemophilia across Europe – a Socioeconomic Survey II” (CHESS II) cross-sectional, retrospective burden of illness study among adults with hereditary haemophilia A or B from eight European countries. HA patients of any severity with no active inhibitors during the 12 months prior to data capture and a completeEQ-5D-5 L response were included. A base GLM model was specified with covariates for demographic and clinical characteristics (age, body mass index, country, employment, HA severity, annual bleeding rate, problem joints, and chronic pain).

**Results:**

Of 381 evaluable patients, 221 (58.0%) had severe HA, 96 (25.2%) had moderate HA, and 64 (16.8%) had mild HA. Among the covariates included in the GLM model and after controlling for haemophilia-related outcomes, a significant association was observed between mild HA and higher EQ-5D-5 L index score (average marginal effects, 0.084; *p* = 0.016) relative to severe HA. Patient country of residence and magnitude of HA-related chronic pain were also associated with significant differences in index scores, with the latter showing a negative relationship with HRQoL outcomes.

**Conclusions:**

Condition severity and chronic pain are significant predictors of HRQoL in PwHA. Durable bleeding protection and effective management of chronic pain have the potential to address unmet treatment needs in this population.

**Supplementary Information:**

The online version contains supplementary material available at 10.1186/s12955-024-02267-6.

## Background

Haemophilia A (HA) is a congenital X-linked bleeding disorder caused by a deficiency or absence of clotting factor VIII (FVIII) [1, 2]. HA has been reported to occur in approximately 1 of every 4,000–5,000 male births, with a recent meta-analysis estimating the prevalence of all-severity HA at birth as 24.6 cases per 100,000 males [2–4].

HA may be classified as mild (endogenous FVIII activity levels > 5–40% of normal), moderate (activity 1–5% of normal), or severe (activity < 1% of normal) [2, 5]. The disorder is characterised by prolonged bleeding following haemostatic challenges (e.g., surgery or minor trauma), with additional risk of spontaneous (non-trauma related) bleeding in patients with severe HA [4, 6]. Most bleeding events occur in the musculoskeletal system, with approximately two-thirds of intra-articular bleeds arising in the major joints (knees, elbows and ankles) and resulting in joint swelling and acute pain [7, 8]. Recurrent intra-articular bleeding can lead to persistent synovial inflammation and haemophilic arthropathy, with increased risk of chronic pain and reduced mobility caused by progressive joint stiffness and deformity. These sequelae ultimately lead to disability and reduced health-related quality of life (HRQoL) for people with HA (PwHA), with accompanying psychosocial burden and significant healthcare resource use [6, 9–12].

Since the 1970s, the mainstay of HA management has been use of intravenous (IV) recombinant or plasma-derived FVIII replacement. For people with moderate and mild HA, infusions are generally administered as an acute treatment during a bleeding event or as short-term prophylaxis prior to an invasive procedure [13, 14]. For people with severe HA or a phenotype characterised by frequent bleeding events, a long-term prophylaxis (LTP) regimen of FVIII replacement, ideally initiated in infancy, is recommended in order to reduce bleed risk and minimise joint damage [2, 15–17]. LTP in HA is a demanding treatment regimen necessitating frequent (≥ 2 per week) infusions [18], which can have a substantial impact on HRQoL and treatment adherence [19, 20]. Breakthrough bleeding episodes remain a risk even for those patients fully adherent to their dosing regimen [21, 22].

Recent clinical development has focused on minimising treatment burden for PwHA through a variety of approaches: increasing the half-life of replacement FVIII (extended half-life [EHL] products) to reduce the frequency of administration; substitution of IV-administered products with subcutaneous non-factor replacement therapies (NFRTs) that permit up to monthly dosing regimens (e.g. emicizumab); and inducing long-lasting endogenous FVIII production, such as with gene therapy, which could eliminate the need for LTP and thus its associated burden [19, 23].

Though the broad implications of treatment burden on HRQoL are coming into focus, there is little research informing the role of HA severity on HRQoL. As haemophilia management improves clinically, it is important to characterise and differentiate the HRQoL of PwHA at varying levels of condition severity, so that informed decisions can be made about the appropriate use of emerging therapies. This analysis aimed to explore the relationship between HRQoL and HA severity and other clinical determinants using real-world data from a cohort of adult PwHA.

## Methods

We developed a generalised linear regression model to explore the relationship between HA severity and patient-reported HRQoL using data from the “Cost of Haemophilia across Europe – a Socioeconomic Survey II” (CHESS II) study.

### Data source and analysis population

CHESS II was a cross-sectional, 12-month retrospective study of the humanistic and economic burden of haemophilia A and B among adults (≥ 18 years) in eight European countries. Data for 1,337 individuals, including 918 PwHA, was collected by means of two questionnaires: a web-based case record form (CRF), completed by the treating haematologist or haemophilia care provider (*n* = 185) and which comprised demographic, clinical and resource use information; and a voluntary “Patient and Public Involvement and Engagement” (PPIE) form for the patient, covering workforce participation and non-medical direct and indirect costs. HRQoL was also captured in *n* = 559 PwH using the EQ-5D-5 L health utility measure [24–26]. To minimise selection bias, physicians were encouraged to recruit the next eligible patients with whom they consulted (up to a maximum of 16), regardless of consultation reason. Recruitment and data collection were carried out between November 2018 and October. Further details on the CHESS II study have been described previously [24, 27]. The analysis set consisted of individuals with mild, moderate, or severe HA and no diagnosis of an active inhibitor during the 12 months prior to data capture.

### Demographics and clinical outcomes

Bleeding events (clinician recorded) included both bleeds (mild pain, minimal swelling, minimal restriction of motion and spontaneous resolution or response within 24 h of treatment) and “major” bleeds (characterised by pain, effusion, swelling, restricted range of motion and failure to respond to treatment within 24 h) experienced in the 12 months prior to data collection. An annual bleeding rate (ABR) was calculated using the sum of all reported bleeding events. Chronic joint disease was captured using a haemophilia-specific “problem joint” classification (defined as joints with chronic pain and/or limited range of movement due to chronic synovitis or arthropathy, with or without persistent bleeding) [28]. HA-related chronic pain was classified by the treating clinician using a 1–4 scale defined as: (1) none (no functional deficit; no analgesic use except with acute haemarthrosis); (2) mild (does not interfere with occupation or activities of daily living (ADL); may require occasional non-narcotic analgesic); (3) moderate (partial or occasional interference with occupation or ADL; use of non-narcotic medications); or (4) severe (interferes with occupation or ADL; requires frequent use of non-narcotic and narcotic medications) [29]. Treatment regimen (% on LTP) was reported descriptively.

### Health-related quality of life: EQ-5D-5 L

The EQ-5D-5 L is a five-item questionnaire comprising five dimensions evaluating mobility, self-care, usual activities, pain/discomfort, and anxiety/depression [30]. Dimensions are scored by the respondent according to their perceived level of impairment on the day of reporting using a 1–5 scale (none; slight; moderate; severe; extreme/unable). Responses are weighted (using weights derived from general population preferences) [31] to convert them onto a 0–1 utility scale, reflecting the respondent’s health status (where 0 represents a “dead” or equivalent state and 1 represents a state of “perfect health”), with values below zero also possible. For this analysis, the UK EQ-5D-5 L–3 L crosswalk was applied using the mapping algorithm developed by Hernandez Alava and colleagues [32] in order to allow for HRQoL for the full analysis sample (including observations from all countries) to be assessed in aggregate. An accompanying visual analogue scale (VAS; EQ-VAS) ranging from 0 (“the worst health you can imagine”) to 100 (“the best health you can imagine”) allows the respondent to indicate their perceived health status [33].

### Statistical analysis

Patient characteristics, clinical outcomes, and HRQoL outcomes (EQ-5D-5 L dimension scores, index score and EQ-VAS) were reported descriptively and compared across severity cohorts. Continuous variables were presented as mean, standard deviation (SD), median and interquartile range (IQR) as appropriate, and normality was assessed using a Shapiro Wilk test. Categorical variables were presented as frequencies and percentages. As none of the assessed variables were normally distributed, statistical significance between severity cohorts was analysed using the Kruskal-Wallis test, confirmed with Dunn’s test.

The marginal effect of HA severity on EQ-5D-5 L index scores was evaluated using a series of generalised linear model (GLM), a more flexible extension of linear regression model [34, 35] that accounts for the nonparametric and bounded nature of the scores [36]. Assessment of covariates for inclusion in a base model included descriptive analyses and evaluation of covariate distribution and multicollinearity, as well as univariate regression analysis to identify variables that had a significant association (*p* < 0.05) with index scores. The log-link function in combination with a Gamma distribution was used to model index scores, which were transformed to a “disutility” index score (nY = 1-Y) to fit the model specification [37, 38]. Distributions were assessed for normality and skewness, and confirmatory analysis of the distribution family was performed with a modified Park test [39]. In addition to the base model (which included age, BMI, employment status, marital status, country, ABR, PJs and chronic pain), two additional exploratory models utilizing a subset of covariates were specified to further improve model fit and performance: Model 2 (excluding marital status) and Model 3 (excluding marital status and comorbidity covariates). Country of residence was included as a covariate to control for any country/cultural factors potentially affecting results. Patient-reported socio-economic status was not available for all participants, therefore employment status and BMI covariates were included as alternative socio-economic indicators [40]. Additional evaluations of Model 3 using the Italian and the Spanish value sets were carried out to ensure the validity of the approach [34, 35].

Model performance was examined, and tests assessed model fit based on deviance, Akaike’s Information Criterion (AIC), Bayesian Information Criterion (BIC) and Root Mean Square Error (RMSE). Model results are presented as the average marginal effect (AME) of HA severity on EQ-5D-5 L index scores. The null hypothesis assumed no significant effect of HA severity on index scores; our alternative hypothesis posited a negative association of index score with HA severity. A significance level of *p* < 0.05 was set to determine the rejection of the null hypothesis. Sensitivity analysis was conducted to test for possible ceiling effects in index score by specifying a model equivalent to Model 3 and excluding observations with a utility value of 1, in order to evaluate both the magnitude of impact on AMEs relative to Model 3 and the overall robustness of our findings, All statistical analyses were conducted using STATA^®^ 17 (StataCorp LLC, College Station, Texas; www.stata.com).

## Results

### Demographics and clinical characteristics

Of the 886 PwHA meeting the inclusion criteria, 381 had complete EQ-5D-5 L responses and were included in the analysis. Among those, 221 (58%) had severe HA, 96 (25.2%) had moderate HA, and 64 (16.8%) had mild HA. Baseline characteristics for each cohort are provided in Table 1. Mean (SD) age was 35.7 (14.4) years, with the largest age group comprised of individuals aged 18–25 years (*n* = 120, 31.5%). Mean BMI was 24.6 (2.5) kg/m^2^; both age and BMI were consistent across severity cohorts. The majority of patient participants were from Italy (*n* = 121, 31.8%) and Spain (*n* = 112, 29.4%), and either in full-time or part-time employment (*n* = 206, 54.9%) or in full-time education (*n* = 66, 17.6%). Mean (SD) ABR was lowest in the mild HA cohort (1.2; 0.9) and highest in the severe cohort (3,8;3.0; *p* < 0.001 versus mild). The distribution of problem joints across cohorts showed larger variability in the severe HA cohort, with a larger proportion with reports of 1 + problem joints(47% severe HA vs. 15.7% mild HA) and with a mean (SD) of 0.8 (1.0]) problem joints compared with 0.2 (0.4; *p* < 0.001 versus severe) in the mild cohort. Approximately two-thirds of individuals experienced some level of HA-related chronic pain, with 19.8% of the moderate cohort and 41.2% of the severe cohort experiencing either moderate or severe chronic pain.

**Table 1:** Patient characteristics

	Haemophilia severity					
	Mild		Moderate	Severe		All
	*n* = 64		*n* = 96	*n* = 221		*n* = 381
**Mean age, years**	34.8 ± 14.8		36.2 ± 15.3	35.8 ± 13.9		35.7 ± 14.40
18–25	21 (32.8%)		30 (31.3%)	69 (31.2%)		120 (31.5%)
26–35	16 (25.0%)		26 (27.1%)	54 (24.4%)		96 (25.2%)
36–50	18 (28.1%)		24 (25.0%)	65 (29.4%)		107 (28.1%)
51–65	5 (7.8%)		8 (8.3%)	27 (12.2%)		40 (10.5%)
66+	4 (6.3%)		8 (8.3%)	6 (2.7%)		18 (4.7%)
**Country**						
Germany	3 (4.7%)		9 (9.4%)	15 (6.8%)		27 (7.1%)
Spain	15 (23.4%)		28 (29.2%)	69 (31.2%)		112 (29.4%)
France	18 (28.1%)		19 (19.8%)	29 (13.1%)		66 (17.3%)
Italy	19 (29.7%)		24 (25.0%)	78 (35.3%)		121 (31.8%)
United Kingdom	9 (14.1%)		16 (16.7%)	30 (13.6%)		55 (14.4%)
**BMI**	23.9 ± 2.3		24.9 ± 2.9	24.7 ± 2.5		24.6 ± 2.5
**Employment status** ^**a**^						
Employed	40 (62.5%)		46 (47.9%)	120 (55.3%)		206 (54.9%)
Not employed due to HA	1 (1.6%)		3 (3.1%)	14 (6.5%)		18 (4.8%)
Not employed – other	8 (12.9%)		23 (24.0%)	37 (17.1%)		68 (18.1%)
Student	13 (21.0%)		17 (17.7%)	36 (16.6%)		66 (17.6%)
Other	0 (0%)		7 (7.3%)	10 (4.6%)		17 (4.5%)
**ABR** (median, IQR)	1.16 ± 0.91 (1, 2)		2.19 ± 3.22 (1, 2)	3.78 ± 2.99 (3, 3)		2.94 ± 3.00 (2, 3)
**Problem joints** (median, IQR)	0.17 ± 0.42 (0, 0)		0.52 ± 0.96 (0, 1)	0.77 ± 1.03 (0, 1)		0.61 ± 0.96 (0, 1)
**Number of problem joints**						
0	54 (84.4%)		65 (67.7%)	117 (52.9%)		236 (61.9%)
1	9 (14.1%)		19 (7.3%)	60 (27.1%)		88 (23.1%)
2	1 (1.6%)		8 (8.3%)	29 (13.1%)		38 (10.0%)
3+	0 (0%)		4 (4.2%)	15 (6.8%)		19 (5.0%)
**Treatment regimen**						
LTP	0 (0%)		0 (0%)	145 (65.6%)		145 (38.1%)
**Chronic pain**						
None	39 (60.9%)		34 (35.4%)	45 (21.7%)		121 (37.8%)
Mild	23 (35.9%)		43 (44.8%)	82 (37.1%)		148 (38.8%)
Moderate	2 (3.0%)		18 (18.8%)	74 (33.5%)		94 (24.7%)
Severe	0 (0%)		1 (1.0%)	17 (7.7%)		18 (4.7%)
**EQ-5D-5 L index score** (median, IQR)	0.85 ± 0.20 (0.89, 0.18)		0.78 ± 0.18 (0.68, 0.26)	0.67 ± 0.26 (0.68, 0.36)		0.73 ± 0.24 (0.75, 0.35)
**EQ-5D-5 L dimension scores**						
Mobility	1.34 ± 0.70		1.58 ± 0.71	2.02 ± 0.98		1.80 ± 0.91
Self-care	1.25 ± 0.67		1.40 ± 0.70	1.69 ± 0.87		1.54 ± 0.82
Usual activities	1.27 ± 0.62		1.51 ± 0.70	1.95 ± 0.94		1.73 ± 0.88
Pain/discomfort	1.50 ± 0.69		1.80 ± 0.78	2.17 ± 0.96		1.96 ± 0.91
Anxiety/depression	1.52 ± 0.84		1.56 ± 0.63	1.91 ± 0.89		1.76 ± 0.84
**EQ-VAS** (median, IQR)	80.65 ± 15.57 (80, 25)		78.0 ± 14.51 (80, 20)	66.81 ± 19.29 (70, 24)		71.93 ± 18.60 (75, 25)

*Note* Results reported as n (%) or mean ± SD unless specified

^a^ Patient-reported employment information was missing for *n* = 6 participants. Proportions are based on non-missing data

*Abbreviations* BMI, Body Mass Index; ABR, Annualised Bleeding Rate; IQR, Inter-Quartile Range; LTP, Long-Term Prophylaxis

### EQ-5D-5 L descriptive analysis

A mean EQ-5D-5 L index score of 0.73 (0.23) was recorded for the full analysis cohort, with a lower mean score for individuals with severe HA (0.67, 0.25), versus both moderate (0.77, 0.18; *P* < 0.001) and mild HA (0.85, 0.19; *P* < 0.001). Nearly one-quarter (23%, *n* = 89) of respondents reported full health (index score equal to 1). Of these, 31% (*n* = 28), 26% (*n* = 23) and 43% (*n* = 38) had mild, moderate and severe HA, respectively, and represented 44%, 24%, and 17% of the respective severity cohorts. Mean EQ-5D-5 L dimension scores increased consistently with condition severity, denoting increased burden (Table 1). EQ-VAS score was also lower among the severe cohort (66.81,19.29) compared with moderate (78.0,14.51; *P* < 0.001) and mild cohorts (80.65,15.57; *p* < 0.001).

Univariate analysis demonstrated statistically significant relationships between EQ-5D-5 L index score and all variables, with the exception of age. FVIII treatment strategy was not included as a covariate because no patients with mild or moderate HA were receiving LTP with FVIII (Table 1). Similarly, treatment adherence, while analysed descriptively, was not included as it was only applicable to individuals receiving LTP.

### EQ-5D-5 L multivariable analysis

Of three multivariable models specified, the model with the most restricted set of covariates (Model 3, Table 2) was found to perform best in terms of fit and efficiency (full model fit and efficiency assessment results are available in Supplementary Table S2 [Supplementary Materials]).

Estimates of the relationships between HA severity, demographics, clinical characteristics and EQ-5D-5 L index score for Model 3 are summarised in Table 2. Mild HA was significantly associated with higher index scores (AME 0.087; *p* = 0.003) relative to severe HA. Chronic pain was significantly associated with lower index scores, with AME − 0.158 (SD 0.022; *P* < 0.001), − 0.239 (0.0439; *P* < 0.001), and − 0.332 (SD 0.01; *P* < 0.001) for mild, moderate, and severe chronic pain, respectively. With respect to demographic characteristics, index scores were significantly lower for the UK and Italy relative to the other respondent countries (AME − 0.161 (SD 0.051; *P* = 0.002) and − 0.099 (SD 0.036; *P =* 0.005), respectively). No notable differences in magnitude or significance of the predictors were observed when comparing across the three models (see Supplementary Table S1 [Supplementary Materials]).

**Table 2:** Relationships of covariates with EQ-5D-5 L index scores (model 3)

		AME ± SE	95% confidence interval
**HA severity**			
Severe		*Reference*	*Reference*
Moderate		0.046 ± 0.029	-0.012, 0.103
Mild		0.087*** ± 0.03	0.029, 0.145
**Age, years**		0.001 ± 0.001	-0.001, 0.003
**BMI**		0.001 ± 0.005	-0.009, 0.011
**Employment status**			
Employed		*Reference*	*Reference*
Not employed due to HA		-0.125 ± 0.079	-0.281, 0.03
Not employed – other		-0.142*** ± 0.044	-0.229, -0.054
Student		0.024 ± 0.03	-0.035, 0.084
Other		-0.011 ± 0.057	-0.122, 0.101
**Country**			
France		*Reference*	*Reference*
Germany		-0.006 ± 0.043	-0.09, 0.078
Spain		-0.037 ± 0.034	-0.104, 0.029
Italy		-0.099*** ± 0.036	-0.17, -0.029
United Kingdom		-0.161*** ± 0.051	-0.26, -0.061
**ABR**		-0.008 ± 0.005	-0.019, 0.003
**Problem joints**		-0.016 ± 0.017	-0.049, 0.017
**Chronic pain**			
None		*Reference*	*Reference*
Mild		-0.158*** ± 0.022	-0.202, -0.115
Moderate		-0.239*** ± 0.039	-0.316, -0.162
Severe		-0.332*** ± 0.1	-0.527, -0.136

*Abbreviations* AME, Average Marginal Effect; HA, Haemophilia A; SE, Standard Error; BMI, Body Mass Index; ABR, Annualised Bleeding Rate

*P < 0.10;**P < 0.05; ***P < 0.01

Sensitivity analysis based on Model 3 was also carried out excluding observations reporting full health (*n* = 89), which confirmed the findings from Model 3 and revealed no differences of note in magnitude, directionality, or statistical significance of coefficients. The additional evaluation of Model 3 using the Italian and Spanish value sets for EQ-5D-5 L revealed no major deviations from the results presented in Table 2. Full model assessments are available in Supplementary Table S3 [Supplementary Materials].

## Discussion

This analysis used real-world data to examine how the severity of HA, as measured by endogenous FVIII activity levels, relates to HRQoL in a European cohort of adult PwHA. After controlling for confounders in a multivariable analysis (including geography, demographic and clinical characteristics), EQ-5D-5 L index scores were found to be higher in patients with mild HA compared with the moderate and severe cohorts, in the context of the minimum clinically important difference associated with EQ-5D index values (0.07), consistent with prior reports where mild or moderate HA were generally found to be associated with higher HRQoL [20, 36–42]. All outcomes were observed to be worse in patients with severe HA. Mean ABR and problem joint frequency were highest for severe HA and declined with decreasing severity, with statistically significant differences between the severe and mild subgroups. Chronic pain was more prevalent in the severe cohort, with the majority of individuals reported to experience at least some level of chronic pain and nearly one-third experiencing moderate to severe chronic pain.

Consistent with previous studies by Holstein et al. and Carrol et al., chronic pain was found to be highly prevalent in this cohort at all levels of condition severity and a significant predictor of lower HRQoL [36, 43]. The detrimental impact of chronic pain on HRQoL has been observed in other chronic conditions, as has the influence of disease-specific outcomes, comorbid conditions and socio-demographic factors [44–49]. Joint disease and higher ABR were not found to be independent predictors of reduced HRQoL in this analysis; however, directionality of effect (i.e., suggestive of a negative effect on HRQoL) was similar to other real-world studies in PwH [36, 43]. History of prophylactic treatment was suggested as a driving factor of HRQoL in haemophilia populations [20, 50–52], however these findings refer to severe PwHA, while the cohort examined here also included mild and moderate PwHA. This emphasises a continued need for upstream management of the precursors to chronic pain in HA, including early intervention with appropriate treatment to minimise bleeding occurrence and proactive rehabilitation of impacted joints. Understanding the relationship between condition severity and HRQoL is critical to accurately characterizing the value of emerging therapies. These findings highlight a statistically significant association of mild HA with increased HRQoL (with respect to severe condition), suggesting a role of novel therapeutic approaches, such as gene therapy, that can reduce treatment burden associated with chronic treatment, while providing durable bleed control. The ultimate goal for such therapies would be an alleviation of the psychosocial and economic burden associated with HA [6, 53–55].

Whilst this study had a relatively large sample size for a rare condition, interpretation of these findings should consider certain inherent limitations. Completion of the PPIE questionnaire (containing the EQ-5D-5L) was voluntary and contingent upon individuals visiting their physicians. Furthermore, the study recruitment method may have resulted in an underrepresentation of individuals with mild and moderate HA who generally engage less frequently with their treating physicians compared to those individuals with severe HA [56, 57]. Both factors suggest that some degree of selection bias may have been present. The cross-sectional design of CHESS II and the EQ-5D-5L’s “today” recall period both limit our ability to assess the longer-term impact of clinical outcomes on health status, as well as any accounting for possible clinical or non-clinical confounders impacting over an extended timeframe. A future analysis using repeated measures could provide additional validity to the findings, further expand on the effect of specific characteristics and outcomes driving HRQoL in this population. The statistically significant differences in HRQoL observed between some countries may be driven by differences in sample sizes across countries (both overall and in terms of condition severity), as well as differences in their health systems, disease management strategies, and other country- and culture-specific factors that may influence both individual behaviours and/or physician management practices. Controlling for country, among other confounders, within the multivariable analysis attempts to address this limitation. Other limitations relate specifically to our outcome of interest. Despite the established sensitivity of the EQ-5D-5L, some studies – including several in HA [58–62] – have reported a ceiling effect in the measure, particularly among healthy and/or young individuals. In the presented analysis, almost one-quarter of respondents gave responses equivalent to ‘full health’ (value of 1 on a 0–1 scale). A relatively large proportion (31.5%) of this cohort were under 26 years of age – a cohort more likely to have retained better joint health and low ABR relative to older PwHA [63]. The results of our sensitivity analysis suggest that the model remains robust to possible ceiling effects in the dependent variable. However, alternative, disease specific HRQoL measures may be better capable of capturing the nuances of HA’s impact among younger PwHA and those with lower levels of functional impairment. The high incidence of respondents with full health in our analysis may also be driven by a phenomenon observed in other studies, in which individuals with severe impairments report high levels of HRQoL; in some cases, health state valuations by these individuals can exceed general population valuations of analogous health states [64–66]. This so-called ‘disability paradox’, often observed in populations with chronic diseases [67], arises from a discordance between an individual’s perceptions of their personal health and their objective health status, and can be influenced by a range of contextual factors that can promote an individual’s adaptation to their disability and deployment of effective coping strategies. In our analysis, the influence of this paradox in EQ-5D-5L responses is likely highest among those with moderate or severe condition, for whom the divergence between health perceptions and health status has potential to be the largest [64–66]. The potential for the presence of ceiling effects and/or disability paradox in the responses used in this analysis could lead to the underestimation of the ‘true burden’ of HA in this cohort and thus warrants further exploration.

## Conclusions

This study provides novel insights into the nature and magnitude of the relationship between condition severity (as a proxy for condition and treatment burden) and HRQoL in PwHA. These findings are consistent after controlling for immediately observable and/or irreversible clinical outcomes (bleeding events, joint damage, and chronic pain). Individuals with mild HA reported the highest HRQoL, with reductions in HRQoL evident as HA severity and level of chronic pain increased. Our findings suggest that increased and consistent protection against bleeding events and HA-related chronic pain may improve HRQoL for PwHA. With the emergence of potentially transformative therapies that are able to provide prolonged and sustained bleeding protection, further research will be needed to advance our understanding of the relationship between HA severity and psychosocial health and outcomes, in the context of an evolving treatment landscape.

### Electronic supplementary material

Below is the link to the electronic supplementary material.

Supplementary Material 1

## Data Availability

The data that support the findings of this study may be available from HCD Economics, Ltd but restrictions apply to the availability of these data, which were used under license for the current study, and so are not publicly available. Data may be available from the authors upon reasonable request and with permission of HCD Economics Ltd.

## Abbreviations

ABR: Annual Bleeding Rate
AIC: Akaike’s Information Criterion
AME: Average Mean Effects
BIC: Bayesian Information Criterion
BMI: Body Mass Index
CRF: Clinical Record Form
FVIII: Clotting Factor VIII
GLM: Generalised Linear Model
HA: Haemophilia A
HRQoL: Health-Related Quality of Life
LTP: Long-Term Prophylaxis
PPIE: Patient and Public Involvement and Engagement
PwHA: People with Haemophilia A
QoL: Quality of Life
RMSE: Root Mean Square Error
SD: Standard Deviation
VAS: Visual Analog Scale
