# Supplementary material for: Exploring the relationship between condition severity and health-related quality of life in people with haemophilia A across Europe: a multivariable analysis of data from the CHESS II study
Source: Health Qual Life Outcomes. 2024 Jul 29;22:58. doi: 10.1186/s12955-024-02267-6 (PMC11288067; doi:10.1186/s12955-024-02267-6)
Supplement: Supplementary file 1 — Supplementary Material 1 [file 12955_2024_2267_MOESM1_ESM.docx]

**Exploring the relationship between condition severity and health-related quality of life in people with haemophilia A across Europe: a multivariate analysis of data from the CHESS II study**

Enrico Ferri Grazzi^1^, Charles Hawes^2^, Charlotte Camp^2^, David Hinds^3^ Jamie O’Hara^1,4^, Tom Burke^1,4^

^1^HCD Economics, Daresbury, UK; ^2^BioMarin Europe, London, UK; ^3^ BioMarin Pharmaceutical, San Rafael CA, USA; ^4^University of Chester, Chester, UK

**Supplementary Materials**

**Table S1. Relationships of covariates with EQ-5D-5L index scores in Models 1, 2**

|  | | | **Model 1** | | | **Model 2** | |
| --- | --- | --- | --- | --- | --- | --- | --- |
|  | | | **AME ± SE** | | **95% confidence interval** | **AME ± SE** | **95% confidence interval** |
| Haemophilia severity | | |  | |  |  |  |
| Severe |  | | Reference | | Reference | Reference | Reference |
| Moderate |  | | 0.038 ± 0.03 | | -0.022, 0.097 | 0.04 ± 0.03 | -0.018, 0.099 |
| Mild |  | | 0.077** ± 0.031 | | 0.017, 0.138 | 0.083*** ± 0.03 | 0.024, 0.143 |
| Age |  | | 0.002 ± 0.001 | | -0.001, 0.004 | 0.001 ± 0.001 | -0.001, 0.003 |
| BMI |  | | 0 ± 0.005 | | -0.011, 0.01 | 0.001 ± 0.005 | -0.01, 0.011 |
| Employment |  | |  | |  |  |  |
| Employed |  | | Reference | | Reference | Reference | Reference |
| Not employed due to haemophilia | | | | -0.106 ± 0.077 | -0.258, 0.046 | -0.124 ± 0.08 | -0.28, 0.033 |
| Not employed – other reasons | | | -0.137*** ± 0.046 | | -0.226, -0.047 | -0.132*** ± 0.044 | -0.219, -0.045 |
| Student | | | 0.021 ± 0.032 | | -0.041, 0.084 | 0.027 ± 0.031 | -0.033, 0.086 |
| Other |  | | -0.003 ± 0.057 | | -0.114, 0.108 | -0.003 ± 0.057 | -0.114, 0.108 |
| Marital Status |  | |  | |  |  |  |
| Single |  | | Reference | | Reference | - | - |
| Married/registered partner | | | -0.023 ± 0.03 | | -0.082, 0.037 | - | - |
| Separated/ Divorced/ widowed | | | -0.101 ± 0.075 | | -0.249, 0.046 | - | - |
| Country |  | |  | |  |  |  |
| France |  | | Reference | | Reference | Reference | Reference |
| Germany |  | | -0.009 ± 0.043 | | -0.094, 0.076 | -0.007 ± 0.043 | -0.092, 0.077 |
| Spain |  | | -0.041 ± 0.034 | | -0.108, 0.027 | -0.037 ± 0.034 | -0.104, 0.03 |
| Italy |  | | -0.098*** ± 0.036 | | -0.169, -0.027 | -0.099*** ± 0.036 | -0.17, -0.028 |
| United Kingdom |  | | -0.163*** ± 0.052 | | -0.264, -0.062 | -0.165*** ± 0.052 | -0.266, -0.064 |
| Coinfection |  | |  | |  |  |  |
| Yes |  | | -0.098 ± 0.072 | | -0.24, 0.044 | -0.081 ± 0.071 | -0.221, 0.058 |
| Musculoskeletal condition | |  |  | |  |  |  |
| Yes |  | | -0.013 ± 0.053 | | -0.118, 0.091 | -0.018 ± 0.053 | -0.121, 0.086 |
| Anaemia |  | |  | |  |  |  |
| Yes |  | | -0.012 ± 0.054 | | -0.118, 0.093 | -0.014 ± 0.053 | -0.118, 0.09 |
| ABR |  | | -0.009 ± 0.006 | | -0.02, 0.002 | -0.008 ± 0.006 | -0.019, 0.003 |
| Problem Joints |  | | -0.014 ± 0.017 | | -0.047, 0.02 | -0.015 ± 0.017 | -0.049, 0.018 |
| Chronic pain |  | |  | |  |  |  |
| None |  | | Reference | | Reference | Reference | Reference |
| Mild |  | | -0.163*** ± 0.023 | | -0.208, -0.118 | -0.16*** ± 0.023 | -0.204, -0.115 |
| Moderate |  | | -0.232*** ± 0.039 | | -0.309, -0.154 | -0.232*** ± 0.04 | -0.309, -0.154 |
| Severe |  | | -0.325*** ± 0.1 | | -0.521, -0.13 | -0.319*** ± 0.099 | -0.512, -0.126 |

Abbreviations: AME, average marginal effects; SE, Standard Error; BMI, body mass index; ABR, annualised bleeding rate

*P<0.10

**P<0.05

***P<0.01

**Table S2. Model performance assessment results**

|  | | **RMSE** | **Deviance** | **AIC** | **BIC** |
| --- | --- | --- | --- | --- | --- |
| **Model** | |  |  |  |  |
| Model 1 |  | 0.621 | 316.09 | -0.928 | -1770.19 |
| Model 2 |  | 0.618 | 317.88 | -0.934 | -1780.25 |
| Model 3 |  | 0.614 | 319.04 | -0.947 | -1796.90 |

Abbreviations: RMSE, root mean squared error; AIC, Akaike information criteria; BIC, Bayesian information criteria.

|  | |  |  |  |  |
| --- | --- | --- | --- | --- | --- |
|  | |  |  |  |  |
|  |  |  |  |  |  |
|  |  |  |  |  |  |
|  |  |  |  |  |  |

| **Table S3. Patient characteristics and EQ-5D-5L valued with Italian and Spanish value sets** | | | | | | |
| --- | --- | --- | --- | --- | --- | --- |
|  |  | **Haemophilia Severity** | | |  | |
|  | **Mild** | | **Moderate** | **Severe** | | **All** |
|  | **n=64** | | **n=96** | **n=221** | | **n=381** |
| **Mean age, years** | 34.8 ± 14.8 | | 36.2 ± 15.3 | 35.8 ± 13.9 | | 35.7 ± 14.40 |
| 18-25 | 21 (32.8%) | | 30 (31.3%) | 69 (31.2%) | | 120 (31.5%) |
| 26-35 | 16 (25.0%) | | 26 (27.1%) | 54 (24.4%) | | 96 (25.2%) |
| 36-50 | 18 (28.1%) | | 24 (25.0%) | 65 (29.4%) | | 107 (28.1%) |
| 51-65 | 5 (7.8%) | | 8 (8.3%) | 27 (12.2%) | | 40 (10.5%) |
| 66+ | 4 (6.3%) | | 8 (8.3%) | 6 (2.7%) | | 18 (4.7%) |
| **Country** |  | |  |  | |  |
| Germany | 3 (4.7%) | | 9 (9.4%) | 15 (6.8%) | | 27 (7.1%) |
| Spain | 15 (23.4%) | | 28 (29.2%) | 69 (31.2%) | | 112 (29.4%) |
| France | 18 (28.1%) | | 19 (19.8%) | 29 (13.1%) | | 66 (17.3%) |
| Italy | 19 (29.7%) | | 24 (25.0%) | 78 (35.3%) | | 121 (31.8%) |
| United Kingdom | 9 (14.1%) | | 16 (16.7%) | 30 (13.6%) | | 55 (14.4%) |
| **BMI** | 23.9 ± 2.3 | | 24.9 ± 2.9 | 24.7 ± 2.5 | | 24.6 ± 2.5 |
| **Employment status^a^** |  | |  |  | |  |
| Employed | 40 (62.5%) | | 46 (47.9%) | 120 (55.3%) | | 206 (54.9%) |
| Not employed due to HA | 1 (1.6%) | | 3 (3.1%) | 14 (6.5%) | | 18 (4.8%) |
| Not employed – other | 8 (12.9%) | | 23 (24.0%) | 37 (17.1%) | | 68 (18.1%) |
| Student | 13 (21.0%) | | 17 (17.7%) | 36 (16.6%) | | 66 (17.6%) |
| Other | 0 (0%) | | 7 (7.3%) | 10 (4.6%) | | 17 (4.5%) |
| **ABR**  (median, IQR) | 1.16 ± 0.91  (1, 2) | | 2.19 ± 3.22  (1, 2) | 3.78 ± 2.99  (3, 3) | | 2.94 ± 3.00  (2, 3) |
| **Problem joints** (median, IQR) | 0.17 ± 0.42  (0, 0) | | 0.52 ± 0.96  (0, 1) | 0.77 ± 1.03  (0, 1) | | 0.61 ± 0.96  (0, 1) |
| **Number of problem joints** |  | |  |  | |  |
| 0 | 54 (84.4%) | | 65 (67.7%) | 117 (52.9%) | | 236 (61.9%) |
| 1 | 9 (14.1%) | | 19 (7.3%) | 60 (27.1%) | | 88 (23.1%) |
| 2 | 1 (1.6%) | | 8 (8.3%) | 29 (13.1%) | | 38 (10.0%) |
| 3+ | 0 (0%) | | 4 (4.2%) | 15 (6.8%) | | 19 (5.0%) |
| **Treatment regimen** |  | |  |  | |  |
| LTP | 0 (0%) | | 0 (0%) | 145 (65.6%) | | 145 (38.1%) |
| **Chronic pain** |  | |  |  | |  |
| None | 39 (60.9%) | | 34 (35.4%) | 45 (21.7%) | | 121 (37.8%) |
| Mild | 23 (35.9%) | | 43 (44.8%) | 82 (37.1%) | | 148 (38.8%) |
| Moderate | 2 (3.0%) | | 18 (18.8%) | 74 (33.5%) | | 94 (24.7%) |
| Severe | 0 (0%) | | 1 (1.0%) | 17 (7.7%) | | 18 (4.7%) |
| **EQ-5D-5L index score** (IT)  (median, IQR) | 0.90 ± 0.17  (0.95, 0.96) | | 0.86 ± 0.17  (0.90, 0.15) | 0.75 ± 0.26  (0.81, 0.27) | | 0.81 ± 0.24  (0.86, 0.22) |
| **EQ-5D-5L index score** (ES)  (median, IQR) | 0.88 ± 0.17  (0.92, 0.16) | | 0.82 ± 0.17  (0.84, 0.24) | 0.72 ± 0.23  (0.74, 0.30) | | 0.77 ± 0.21  (0.80, 0.28) |
| **EQ-5D-5L dimension scores** |  | |  |  | |  |
| Mobility | 1.34 ± 0.70 | | 1.58 ± 0.71 | 2.02 ± 0.98 | | 1.80 ± 0.91 |
| Self-care | 1.25 ± 0.67 | | 1.40 ± 0.70 | 1.69 ± 0.87 | | 1.54 ± 0.82 |
| Usual activities | 1.27 ± 0.62 | | 1.51 ± 0.70 | 1.95 ± 0.94 | | 1.73 ± 0.88 |
| Pain/discomfort | 1.50 ± 0.69 | | 1.80 ± 0.78 | 2.17 ± 0.96 | | 1.96 ± 0.91 |
| Anxiety/depression | 1.52 ± 0.84 | | 1.56 ± 0.63 | 1.91 ± 0.89 | | 1.76 ± 0.84 |
| **EQ-VAS**  (median, IQR) | 80.65 ± 15.57  (80, 25) | | 78.0 ± 14.51  (80, 20) | 66.81 ± 19.29  (70, 24) | | 71.93 ± 18.60  (75, 25) |
| Notes: Results reported as n (%) or mean ± SD unless specified.  **^a^** Patient-reported employment information was missing for n=6 participants. Proportions are based on non-missing data.  Abbreviations: BMI, body mass index; ABR, annualised bleeding rate; IQR, inter-quartile range; LTP, long-term prophylaxis; IT, Italy; ES, Spain. | | | | | | |

**Table S3. Relationships of covariates with EQ-5D-5L index scores in Model 3 evaluated with the Italian and Spanish value sets**

|  | | **Model 3 (IT)** | | | **Model 3 (ES)** | |
| --- | --- | --- | --- | --- | --- | --- |
|  | | **AME ± SE** | | **95% confidence interval** | **AME ± SE** | **95% confidence interval** |
| Haemophilia severity | |  | |  |  |  |
| Severe |  | Reference | | Reference | Reference | Reference |
| Moderate |  | -0.011, 0.093 | | -0.011, 0.093 | 0.04 ± 0.029 | 0.04 ± 0.029 |
| Mild |  | 0.075*** ± 0.026 | | 0.024, 0.126 | 0.086*** ± 0.028 | 0.032, 0.14 |
| Age |  |  | |  |  |  |
| BMI |  |  | |  |  |  |
| Employment |  |  | |  |  |  |
| Employed |  | Reference | | Reference | Reference | Reference |
| Not employed due to haemophilia | | | -0.111 ± 0.075 | -0.258, 0.036 | -0.095 ± 0.075 | -0.241, 0.051 |
| Not employed – other reasons | | -0.148*** ± 0.046 | | -0.237, -0.058 | -0.132*** ± 0.045 | -0.22, -0.044 |
| Student | | 0.023 ± 0.025 | | -0.027, 0.073 | 0.024 ± 0.029 | -0.032, 0.081 |
| Other | | -0.024 ± 0.054 | | -0.129, 0.081 | -0.03 ± 0.061 | -0.149, 0.089 |
| Country |  |  | |  |  |  |
| France |  | Reference | | Reference | Reference | Reference |
| Germany |  | -0.011 ± 0.035 | | -0.08, 0.057 | 0.005 ± 0.04 | -0.074, 0.084 |
| Spain |  | -0.042 ± 0.028 | | -0.097, 0.013 | -0.031 ± 0.033 | -0.096, 0.034 |
| Italy |  | -0.098*** ± 0.031 | | -0.159, -0.038 | -0.08*** ± 0.035 | -0.149, -0.012 |
| United Kingdom |  | -0.141*** ± 0.046 | | -0.232, -0.05 | -0.14*** ± 0.051 | -0.239, -0.041 |
| ABR |  | -0.007 ± 0.005 | | -0.017, 0.003 | -0.008 ± 0.005 | -0.019, 0.003 |
| Problem Joints |  | -0.017 ± 0.016 | | -0.048, 0.013 | -0.017 ± 0.017 | -0.049, 0.016 |
| Chronic pain |  |  | |  |  |  |
| None |  | Reference | | Reference | Reference | Reference |
| Mild |  | -0.112*** ± 0.019 | | -0.149, -0.076 | -0.144*** ± 0.022 | -0.187, -0.101 |
| Moderate |  | -0.193*** ± 0.035 | | -0.262, -0.124 | -0.211*** ± 0.038 | -0.285, -0.136 |
| Severe |  | -0.271*** ± 0.095 | | -0.457, -0.085 | -0.269*** ± 0.093 | -0.451, -0.087 |

Abbreviations: IT, Italy; ES, Spain; AME, average marginal effects; SE, Standard Error; BMI, body mass index; ABR, annualised bleeding rate

*P<0.10

**P<0.05

***P<0.01
